# The impact of high total cholesterol and high low-density lipoprotein on avascular necrosis of the femoral head in low-energy femoral neck fractures

**DOI:** 10.1186/s13018-017-0532-0

**Published:** 2017-02-17

**Authors:** Xianshang Zeng, Ke Zhan, Lili Zhang, Dan Zeng, Weiguang Yu, Xinchao Zhang, Mingdong Zhao, Zhicheng Lai, Runzhen Chen

**Affiliations:** 1 Department of Orthopedics, The First Affiliated Hospital of Sun Yat-sen University, Huangpu East Road No. 183, Huangpu District, Guangzhou, 510700 Guangdong China; 2 Department of Anesthesiology, The First Affiliated Hospital of Sun Yat-sen University, Huangpu East Road No. 183, Huangpu District, Guangzhou, 510700 Guangdong China; 3 Ultrasonography Department, The First Affiliated Hospital of Sun Yat-sen University, Huangpu East Road No. 183, Huangpu District, Guangzhou, 510700 Guangdong China; 4 Department of Orthopaedics, Jinshan Hospital, Fudan University, Longhang Road No. 1508, Jinshan District, 201508 Shanghai, China; 5 Zhongshan School of Medical, Sun Yat-sen University, Zhongshan second road 74th, Yuexiu District, Guangzhou City, 510080 Guangdong Province China

**Keywords:** Avascular necrosis of the femoral head, Total cholesterol, Low-density lipoprotein, Femoral neck fracture, Harris Hip Score

## Abstract

**Background:**

Avascular necrosis of the femoral head (AVNFH) typically constitutes 5 to 15% of all complications of low-energy femoral neck fractures, and due to an increasingly ageing population and a rising prevalence of femoral neck fractures, the number of patients who develop AVNFH is increasing. However, there is no consensus regarding the relationship between blood lipid abnormalities and postoperative AVNFH. The purpose of this retrospective study was to investigate the relationship between blood lipid abnormalities and AVNFH following the femoral neck fracture operation among an elderly population.

**Methods:**

A retrospective, comparative study was performed at our institution. Between June 2005 and November 2009, 653 elderly patients (653 hips) with low-energy femoral neck fractures underwent closed reduction and internal fixation with cancellous screws (Smith and Nephew, Memphis, Tennessee). Follow-up occurred at 1, 6, 12, 18, 24, 30, and 36 months after surgery. Logistic multi-factor regression analysis was used to assess the risk factors of AVNFH and to determine the effect of blood lipid levels on AVNFH development. Inclusion and exclusion criteria were predetermined to focus on isolated freshly closed femoral neck fractures in the elderly population. The primary outcome was the blood lipid levels. The secondary outcome was the logistic multi-factor regression analysis.

**Results:**

A total of 325 elderly patients with low-energy femoral neck fractures (AVNFH, *n* = 160; control, *n* = 165) were assessed. In the AVNFH group, the average TC, TG, LDL, and Apo-B values were 7.11 ± 3.16 mmol/L, 2.15 ± 0.89 mmol/L, 4.49 ± 1.38 mmol/L, and 79.69 ± 17.29 mg/dL, respectively; all of which were significantly higher than the values in the control group. Logistic multi-factor regression analysis showed that both TC and LDL were the independent factors influencing the postoperative AVNFH within femoral neck fractures.

**Conclusions:**

This evidence indicates that AVNFH was significantly associated with blood lipid abnormalities in elderly patients with low-energy femoral neck fractures. The findings of this pilot trial justify a larger study to determine whether the result is more generally applicable to a broader population.

## Background

Avascular necrosis of the femoral head (AVNFH) is usually considered to be related to reduced blood flow as a consequence of a surgical approach and mostly occurs in patients over the age of 50 who are admitted with low-energy femoral neck fractures [1–3]. The precise pathogenesis of AVNFH remains unclear. Hyperlipidaemia involvement in the pathogenesis of AVNFH has been proposed based on human and animal studies. It should be primarily considered to be related to an interruption of the vascular supply to the bone leading to bone ischaemia and cellular necrosis [4–6]. Previous studies have confirmed that the excessive use of cortical hormones induces AVNFH and lipid metabolism disorders [5, 7, 8]. However, to our knowledge, no detailed description of AVNFH has been reported in the literature [4, 9].

Whether dysregulated lipid metabolism was related to the occurrence of AVNFH after surgery has rarely been reported [10, 11]. In addition, the reported incidence of postoperative AVNFH in different series has varied greatly [12, 13].

The purpose of this retrospective study was to investigate the relationship between blood lipid abnormalities and AVNFH following a femoral neck fracture operation among the elderly population. Our hypothesis was that AVNFH was significantly associated with blood lipid abnormalities in elderly patients with low-energy femoral neck fractures.

## Methods

### General data

This study was reviewed and approved by the review board of the First Affiliated Hospital at Sun Yat-sen University, Guangzhou, China, and an exemption for informed consent was obtained from our investigational ethical review board. The study was conducted in compliance with the provisions of the Declaration of Helsinki and EN 540.

Between June 2005 and November 2009, 653 patients with an isolated fresh femoral neck fracture (653 hips) undergoing closed reduction and internal fixation with cancellous screws (Smith and Nephew, Memphis, Tennessee) were identified from our orthopaedic trauma database. WY performed the clinical investigation of the patients. All surgeries were finished at our institution by senior orthopaedists (WY, XCZ, XZ, and KZ). The surgical procedures were based on standard protocols for cancellous screws, as recommended by device manufacturers and as described previously by Nagi et al. [1]. Fractures were assessed for Garden classification. Follow-up occurred at 1, 6, 12, 18, 24, 30, and 36 months after surgery. The study was also to evaluate the level and determinants of change in physical activity during the follow-up period. Obvious dysfunction would be excluded. Harris Hip Score (HHS) was used for functional evaluation during examination. A retrospective evaluation of the clinical data and radiographic information was performed at each visit.

### Inclusion and exclusion criteria

Inclusion criteria included an isolated freshly closed femoral neck fracture, age ranging from 50–94 years, the ability to walk independently either without assistance or with auxiliary equipment before the fracture, low-energy femoral neck fractures, and no chronic illness (chronic heart failure, chronic kidney disease, chronic obstructive pulmonary disease, cancer) or major surgery contraindications. Exclusion criteria included age outside the inclusion range, <37 months of follow-up, alcohol abuse, long-term use of hormone drugs, pre-existing femoral head necrosis, multiple traumatic injuries, malabsorption syndrome, metabolic abnormalities, hypertension, developmental dysplasia of the hip (DDH), severe arthrosis/arthritis, severe comorbidities, any diseases affecting the blood supply to the femoral head, bed-ridden status, an American Society of Anesthesiologists (ASA) score of V, language barrier, mental retardation, hemiplegia, or incomplete preoperative data.

### Diagnostic criteria

The diagnostic criteria were as follows: if the X-ray is still clearly showing a visible fracture line 6 months or longer after surgery in conjunction with clinical symptoms, the fracture was defined as non-union. If the X-ray, computed tomography (CT), or magnetic resonance imaging (MRI) scan displayed changes of radionuclide imaging or femoral density, including cystic degeneration, hardening, or uneven density, the patient was diagnosed with AVNFH. Mechanical failure was defined as either a loss of alignment of at least 10° or shortening of at least 2 cm. Deep surgical site infections and reoperations were additionally quantified.

### Detection index

Approximately 2 mL of preoperative fasting venous blood was centrifuged to obtain serum. The Hitachi 7000 automatic biochemical analyzer tested the blood lipid levels, including triglyceride (TG), total cholesterol (TC), low-density lipoprotein (LDL), high-density lipoprotein (HDL), apolipoprotein-B (Apo-B), and apolipoprotein-A1 (Apo-A1).

### Statistical analysis

All continuous data were expressed as the mean ± standard deviation (SD), and the ratio data were expressed as *N* and %; all these data were analysed using the Wilcoxon rank sum test (Mann–Whitney *U* test). Quantitative variables were analysed using the two-tailed Student’s *t* test, and categorical variables were analysed by the *χ* ^2^ test or Fisher’s exact test where appropriate. Multi-factor logistic regression analysis was used to analyse the different influencing factors. Two-tailed *P* values <0.05 were deemed statistically significant. In principle, the *χ* ^2^ test was employed. However, the Fisher’s exact test was used for the analysis of the cases in which the expected frequency was not attained. The software SPSS (version 22.0.0, SPSS Inc., Chicago, Illinois, IBM, New York, NY) was used to analyse the data.

## Results

### General data comparison

**Fig. 1:**
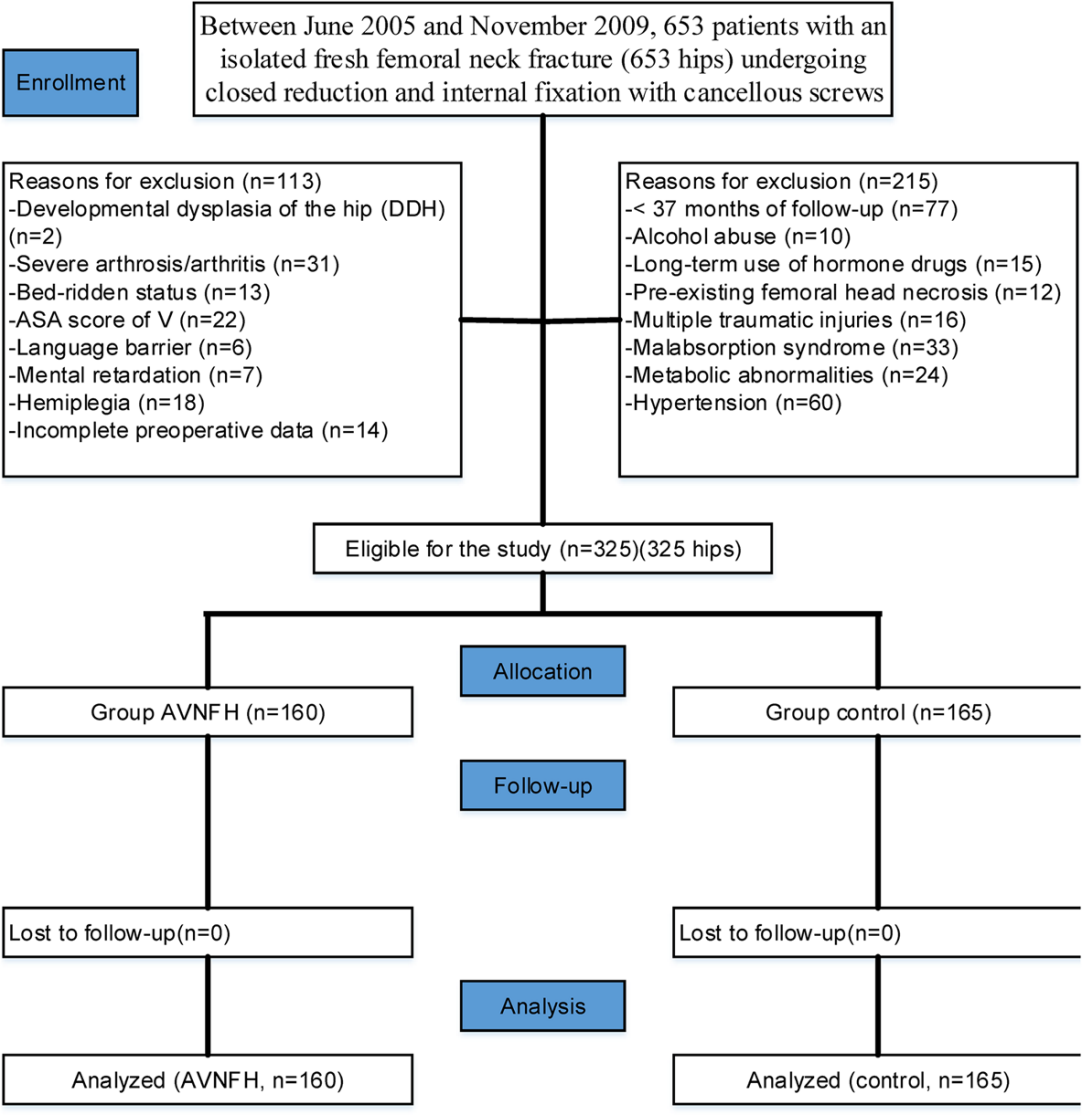
Flow diagram demonstrating methods for identification of studies to investigate the relationship between blood lipid abnormalities and AVNFH following the femoral neck fracture operation among an elderly population

There were 328 of 653 patients (50.2%) excluded based on inclusion and exclusion criteria, leaving 325 patients with a mean age of 74 years (range 50–94 years) who met the inclusion criteria and were available for analysis (two groups: AVNFH [*n* = 160] and control [*n* = 165], Fig. 1). The average body mass index (BMI) was 25.4 (range 14.0–38.0). There were 143 right hips and 182 left hips. The gender distribution was 43.7% male and 56.3% female. The patient demographics are shown in Table 1. All patients had successful operations, including 160 cases of postoperative AVNFH patients, accounting for 49.2%. There were no meaningful differences in gender, age, ASA scale, fracture lateralization, BMI, femoral neck bone mineral density (FNBMD), Garden classification, or preoperative blood lipid levels between the groups (*P* > 0.05) (Tables 1 and 2).

**Table 1:** Patient demographics in the two groups

Variable	AVNFH (*n* = 160)	Control (*n* = 165)	*P* value
Age (years)	75.2 ± 13.37	73.0 ± 11.97	0.12^*a^
Sex (M:F)	68:92	74:91	0.67^*b^
ASA scale, no.			0.22^*c^
I	23	18	
II	41	37	
III	67	76	
IV	29	34	
V	0	0	
Laterality (L/R)	93/67	89/76	0.45^*b^
BMI (kg/m^2^)	26.2 ± 5.62	24.6 ± 6.50	0.19^*a^
FNBMD	2.3 ± 0.91	2.4 ± 1.06	0.09^*a^
Garden classification (no.)			0.15^*c^
I	13	23	
II	35	34	
III	43	48	
IV	69	60	

*AVNFH* avascular necrosis of the femoral head, *ASA* American Society of Anesthesiologists, *BMI* body mass index, *FNBMD* femoral neck bone mineral density

^*^No statistically significant values

^a^Analysed using an independent samples *t* test

^b^Analysed using the chi-square

^c^Analysed using the Mann–Whitney test

**Table 2:** Preoperative comparison of average blood lipid levels between the two groups

Variable	AVNFH (*n* = 160)	Control (*n* = 165)	*P* value
TC (mmol/L)	4.53 ± 0.37	4.48 ± 0.33	0.15^*a^
TG (mmol/L)	1.64 ± 0.42	1.59 ± 0.53	0.32^*a^
HDL (mmol/L)	2.43 ± 0.63	2.50 ± 0.24	0.17^*a^
LDL (mmol/L)	3.02 ± 0.36	2.97 ± 0.14	0.94^*a^
Apo-A1 (mg/dL)	102.46 ± 16.93	105.41 ± 21.39	0.17^*a^
Apo-B (mg/dL)	57.32 ± 7.40	55.50 ± 11.77	0.10^*a^

*AVNFH* avascular necrosis of the femoral head, *TC* total cholesterol, *TG* triglyceride, *HDL* high-density lipoprotein, *LDL* low-density lipoprotein, *Apo-A1* apolipoprotein A1, *Apo-B* apolipoprotein-B

^*^No statistically significant values

^a^Analysed using an independent samples *t* test

### Comparison of fracture treatment

There were no between-group significant differences in the combined Garden index and HHS (*P* > 0.05). At an average follow-up of 42 months (range 37–46 months), 57 mechanical failures (17.5%) occurred. The mean time to diagnosis of AVNFH was 32 months (range 10–38), with 12 mechanical failures occurring within 8 weeks after surgery. No significant differences were observed in terms of reduction mode, Garden index, operation interval, and weight-bearing activity time (Table 3).

**Table 3:** Comparison of the treatment of patients with femoral neck fractures between the two groups

Variable	AVNFH (*n* = 160)	Control (*n* = 165)	*P* value
HHS	72.3 ± 11.25	75.2 ± 9.4	0.01^*a^
Garden index			0.57^*b^
I	42	46	
II	56	52	
III	44	34	
IV	18	33	
Injury operation interval			0.48^*b^
<24 h	26	32	
24–48 h	45	58	
48–72 h	53	35	
>72 h	31	40	
Weight-bearing activity time (<8 months/≥8 months)	37/123	49/116	0.18^*c^
Mechanical failure	14.4% (23/160)	20.6% (34/165)	0.14^*c^

*AVNFH* avascular necrosis of the femoral head, *HHS* Harris Hip Score

^*^No statistically significant values

^a^Analysed using an independent samples *t* test

^b^Analysed using the Mann–Whitney test

^c^Analysed using the chi-square test

### Between-group comparisons of the average blood lipid levels

Follow-up occurred at 1, 6, 12, 18, 24, 30, and 36 months after surgery. In the AVNFH group, the TC, TG, LDL, and Apo-B levels were 7.11 ± 3.16 mmol/L, 2.15 ± 0.89 mmol/L, 4.49 ± 1.38 mmol/L, and 79.69 ± 17.29 mg/dL, respectively, which were significantly higher than those of the control group, while HDL and Apo-A1 levels were 1.41 ± 0.43 mmol/L and 114.96 ± 19.85 mg/dL, respectively, which were significantly lower than those of the control group. All of these differences were statistically significant (*P* = 0.00) (Table 4).

**Table 4:** Postoperative comparison of average blood lipid levels between the two groups

Variable	AVNFH (*n* = 160)	Control (*n* = 165)	*P* value
TC (mmol/L)	7.11 ± 3.16	5.69 ± 1.45	0.00^*a^
TG (mmol/L)	2.15 ± 0.89	1.79 ± 0.65	0.00^*a^
HDL (mmol/L)	1.41 ± 0.43	2.12 ± 0.73	0.00^*a^
LDL (mmol/L)	4.49 ± 1.38	3.07 ± 0.69	0.00^*a^
Apo-A1 (mg/dL)	114.96 ± 19.85	136.13 ± 28.64	0.00^*a^
Apo-B (mg/dL)	79.69 ± 17.29	72.81 ± 13.59	0.00^*a^

*AVNFH* avascular necrosis of the femoral head, *TC* total cholesterol, *TG* triglyceride, *HDL* high-density lipoprotein, *LDL* low-density lipoprotein, *Apo-A1* apolipoprotein A1, *Apo-B* apolipoprotein-B

^*^Statistically significant values

^a^Analysed using an independent samples *t* test

### Logistic multi-factor regression analysis of AVNFH

Logistic multi-factor regression analysis exhibited that in addition to the traditional common indicators, TC and LDL were the independent factors that influenced postoperative AVNFH in patients with a femoral neck fracture (Table 5).

**Table 5:** Logistic regression analysis of factors was applied to identify variables independently associated with AVNFH following femoral neck fractures

Influence factors	*β*	SE	OR	95% CI	*χ* ^2^	*P* value
TC	0.762	0.392	1.23	1.10~2.43	3.29	0.002^*^
TG	2.112	0.751	6.49	1.08~4.17	21.36	0.102
HDL	1.855	0.554	4.78	2.16~5.40	5.04	0.290
LDL	1.484	0.603	4.41	1.33~6.69	9.37	0.001^*^
Apo-A1	0.448	0.835	9.35	1.26~8.85	4.99	0.080
Apo-B	0.566	0.676	5.41	1.33~6.04	21.02	0.107

*TC* total cholesterol, *TG* triglyceride, *HDL* high-density lipoprotein, *LDL* low-density lipoprotein, *Apo-A1* apolipoprotein A1, *Apo-B* apolipoprotein-B, *SE* standard error, *OR* odds ratio, *CI* confidence interval

^*^Statistically significant values

## Discussion

AVNFH is a pathological process of bone cells, haematopoietic bone marrow cells, and fat cell necrosis caused by partial or complete ischaemia of the femoral head due to different reasons [14, 15]. AVNFH is divided into two categories (traumatic and non-traumatic). In recent years, because of an ageing population and an increase in traffic accidents and other traumatic events, the incidence of femoral neck fracture has been increasing year after year [16]. Although the healing rate of femoral neck fracture was significantly increased with the constant updating of internal fixation techniques and materials, the incidence of AVNFH failed to decrease accordingly and remained between 10 and 30% [2, 17]. The incidence of AVNFH in this study was 24.5%, which confirmed those reports. Therefore, how to identify high-risk patients early to minimize the incidence of AVNFH following femoral neck fracture has become an important issue in the field of orthopaedics.

Numerous studies have indicated that dysregulation of lipid metabolism may be one of the most important contributors in AVNFH [7, 18, 19]. Mielants noticed that non-traumatic AVNFH was associated with increased serum lipoprotein and TG levels in patients [20]. Iio reported 103 AVNFH patients, of whom 69 had increased cholesterol and TG [21]. In an experimental study of sex hormones in a femoral head necrosis model, it was found that the first change observed was increased blood fat levels, and the second change occurred in the bone. Some researchers found that serum TG and femoral neck bone density were positively correlated in postmenopausal women [2, 3, 6]. The current study also found that the incidence of AVNFH was significantly higher in the patients with hyperlipidaemia compared to a normal group after the reduction of femoral neck fracture. This difference was statistically significant (*P* < 0.05). Logistic multi-factor regression analysis showed that both TC and LDL were independent risk factors of AVNFH. The results of a study by Okpala et al. were consistent with the conclusions of our study showing that the TC and LDL levels in the AVNFH group were significantly higher than those in the control group, and they also believed that TC and LDL could be considered as independent factors and diagnostic criteria for AVNFH [7].

Regarding the pathogenic mechanism of AVNFH induced by the dysregulation of lipid metabolism, the present study suggested that on the one hand, hyperlipidaemia damages vascular endothelial cells to create pro-thrombotic conditions: the ability of vascular endothelial cells to synthesize nitric oxide (NO) decreases resulting in the dysfunction of vascular contraction and relaxation, which affects bone microcirculation [13, 18, 22]. On the other hand, hyperlipidaemia leads to the formation of fat emboli in the peripheral blood, causing bone microvascular obstructions, increasing the intraosseous pressure, and further aggravating the dysfunction of bone microcirculation [10, 23]. In addition, adipocyte hypertrophy, fat accumulation, and fatty marrow in the femoral head increases bone marrow microcirculation pressure, which contributes to the ischaemia, hypoxia, metabolic disorders, and edema observed in bone marrow tissue, resulting in secondary intracranial pressure increases and further aggravating ischaemia and hypoxia. This creates a vicious cycle, eventually resulting in consequences such as bone dystrophy or bone necrosis [10, 13, 24]. Increased blood viscosity, decreased erythrocyte deformability, and microcirculation congestion also damages the blood supply to the femoral neck fracture site after surgery, which contributes to the relative ease of the development of ischaemic necrosis of the femoral head [25, 26].

There are several limitations to our study. First, a small sample size may have introduced bias. However, the focus of our study is to assess an area that has not been studied extensively in the literature. Second, because this is a retrospective study with problems that are inherent to this methodology, patient- and surgeon-related confounders may have existed. Third, we may not have addressed all potential confounding variables in our analyses. Despite these limitations, this analysis presents long-term follow-up results and is the first to evaluate covariates. A prospective randomised study is needed to assess the relationship between blood lipid abnormalities and AVNFH following the femoral neck fracture operation among an elderly population.

## Conclusions

TC and LDL can be considered as diagnostic criteria for AVNFH after surgical repair of femoral neck fracture. Early intervention in patients with dyslipidaemia may be implemented to prevent or delay the occurrence and development of AVNFH in the early stages.

## Abbreviations

Apo-A1: Apolipoprotein-A1
Apo-B: Apolipoprotein-B
ASA: American Society of Anaesthesiologists
AVNFH: Avascular necrosis of femoral head
BMI: Body mass index
CCD: Collodiaphyseal angle
CD: Computed tomography
DDH: Developmental dysplasia of the hip
FNBMD: Femoral neck bone mineral density
HDL: High-density lipoprotein
HHS: Harris Hip Score
LDL: Low-density lipoprotein
MRI: Magnetic resonance imaging
NO: Nitric oxide
SD: Standard deviation
TC: Total cholesterol
TG: Triglyceride
